# Detection of Anterior and Posterior Glenoid Bone Defects After Reverse Shoulder Arthroplasty With Tomosynthesis in a Pig Model

**DOI:** 10.7759/cureus.103625

**Published:** 2026-02-14

**Authors:** Yoshihiro Hirakawa, Tomoya Manaka, Yoichi Ito, Katsumasa Nakazawa, Yukihide Minoda, Koichi Ichikawa, Jin Nagasawa, Hidetomi Terai

**Affiliations:** 1 Department of Orthopaedic Surgery, Ishikiriseiki Hospital, Higashiōsaka, JPN; 2 Department of Orthopaedic Surgery, Osaka Metropolitan University Graduate School of Medicine, Osaka, JPN; 3 Department of Orthopaedic Surgery, Ito Clinic, Osaka Shoulder Center, Matsubara, JPN; 4 Department of Orthopaedic Surgery, Sano Memorial Hospital, Izumisano, JPN

**Keywords:** bone defect, ct (computed tomography) imaging, glenoid bone defect, periprosthetic bone defect, reverse shoulder arthroplasty, scapular notching, tomosynthesis

## Abstract

Background

Scapular notching is a well-recognized complication of reverse shoulder arthroplasty (RSA) and is typically identified at the inferior scapular neck on conventional radiographic evaluation. However, anterior and posterior glenoid bone defects around the baseplate may be difficult to detect using standard imaging modalities, including radiographic evaluation or computed tomography (CT). Tomosynthesis provides sectional imaging with reduced metal artifact and may improve the detection of region-specific glenoid bone defects after RSA.

Methods

The scapula of a slaughter pig was used in this study. Delta XTEND® implants (DePuy, Warsaw, IN, USA) were used to create four models: anterior, posterior, and inferior glenoid bone defect models, and an intact control model. Images obtained by radiographic evaluation, CT, and tomosynthesis were independently reviewed by 12 orthopedic surgeons to assess the presence or absence of glenoid bone defects. Detection sensitivity and specificity were calculated for each imaging modality.

Results

For anterior and posterior glenoid bone defect models, the sensitivities and specificities of radiographic evaluation were 25%/100% and 8.3%/94.4%, respectively. CT demonstrated sensitivities and specificities of 25%/86.1% and 33.3%/86.1%, respectively. In contrast, tomosynthesis demonstrated a sensitivity of 83.3% and a specificity of 94.4% for both anterior and posterior glenoid bone defects. The sensitivity of tomosynthesis was markedly higher than that of radiographic evaluation and CT.

Conclusions

In this controlled ex vivo model, tomosynthesis enabled improved detection of anterior and posterior glenoid bone defects compared with conventional radiography and CT. Although these findings are limited to an experimental setting, tomosynthesis may serve as a useful adjunctive imaging modality for characterizing region-specific glenoid bone defects after RSA.

## Introduction

Reverse shoulder arthroplasty (RSA) has become widely adopted for the management of complex shoulder conditions, including irreparable rotator cuff tears, failed anatomic shoulder arthroplasty, and fracture sequelae [1,2]. Numerous studies have demonstrated reliable improvements in pain and function following RSA. Consequently, the indications for RSA have expanded to include younger patients owing to its favorable long-term clinical outcomes and enhanced deltoid function [3]. However, on long-term follow-up, RSA-specific complications such as scapular notching have been observed [4].

Scapular notching occurs primarily due to repetitive mechanical contact between the humeral component and the inferior scapular neck during arm adduction [5]. Although some reports have found no clear association between scapular notching and clinical outcomes at mid-term follow-up, other studies have demonstrated a negative impact of notching on long-term results [6,7].

Inferior glenoid bone defects caused by scapular notching can usually be detected using standard radiographic evaluation, and careful monitoring is required to avoid overlooking this complication. Contact between the humeral liner and the glenoid component may occur during internal and external rotation of the humerus, and repetitive contact may result in anterior and posterior glenoid bone defects in a manner similar to the mechanism of inferior scapular notching [5]. However, anterior and posterior scapular notching is difficult to detect using standard anteroposterior (AP) radiographic views of the shoulder. Furthermore, glenoid bone loss adjacent to the baseplate or screws may not be adequately visualized on computed tomography (CT) because of metal artifacts.

In recent years, the usefulness of tomosynthesis for detecting glenoid bone defects after total joint replacement surgery has been reported [8]. Since tomosynthesis can acquire images that are less susceptible to metal artifacts and can construct thin slices of the object, it may detect glenoid bone defects in the anterior or posterior direction, which are difficult to detect with radiographic evaluation using AP views or CT.

Therefore, this study aimed to compare the diagnostic performances and detection sensitivity and specificity of radiographic evaluation, CT, and tomosynthesis for anterior and posterior glenoid bone defects around the baseplate of RSA using a pig model.

## Materials and methods

**Figure 1:**
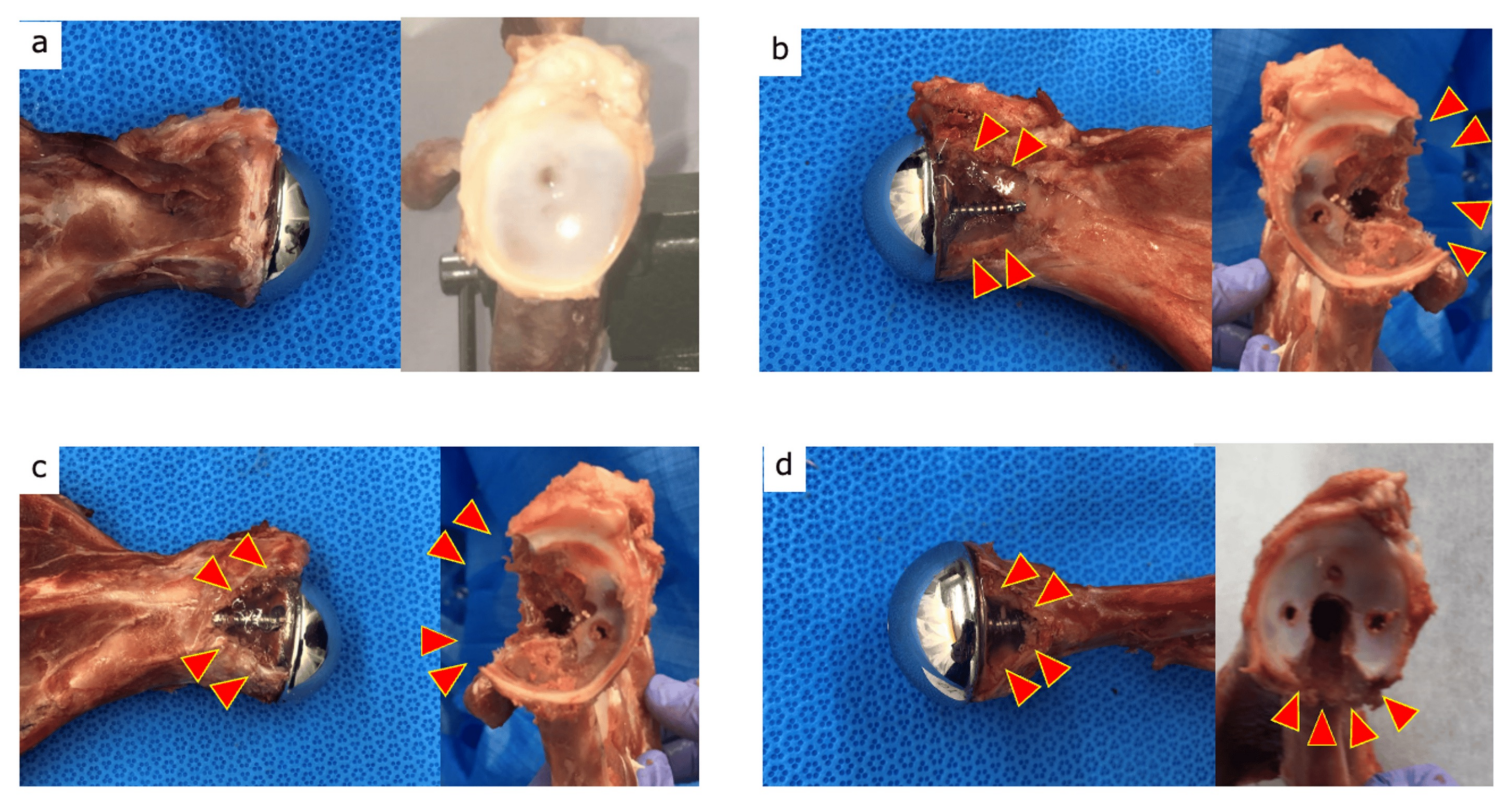
Scapular bone models. Three bone glenoid bone defect models were created until the closest screw was completely exposed. a. control bone, b. anterior glenoid bone defect, c. posterior glenoid bone defect, and d. inferior glenoid bone defect.

**Figure 2:**
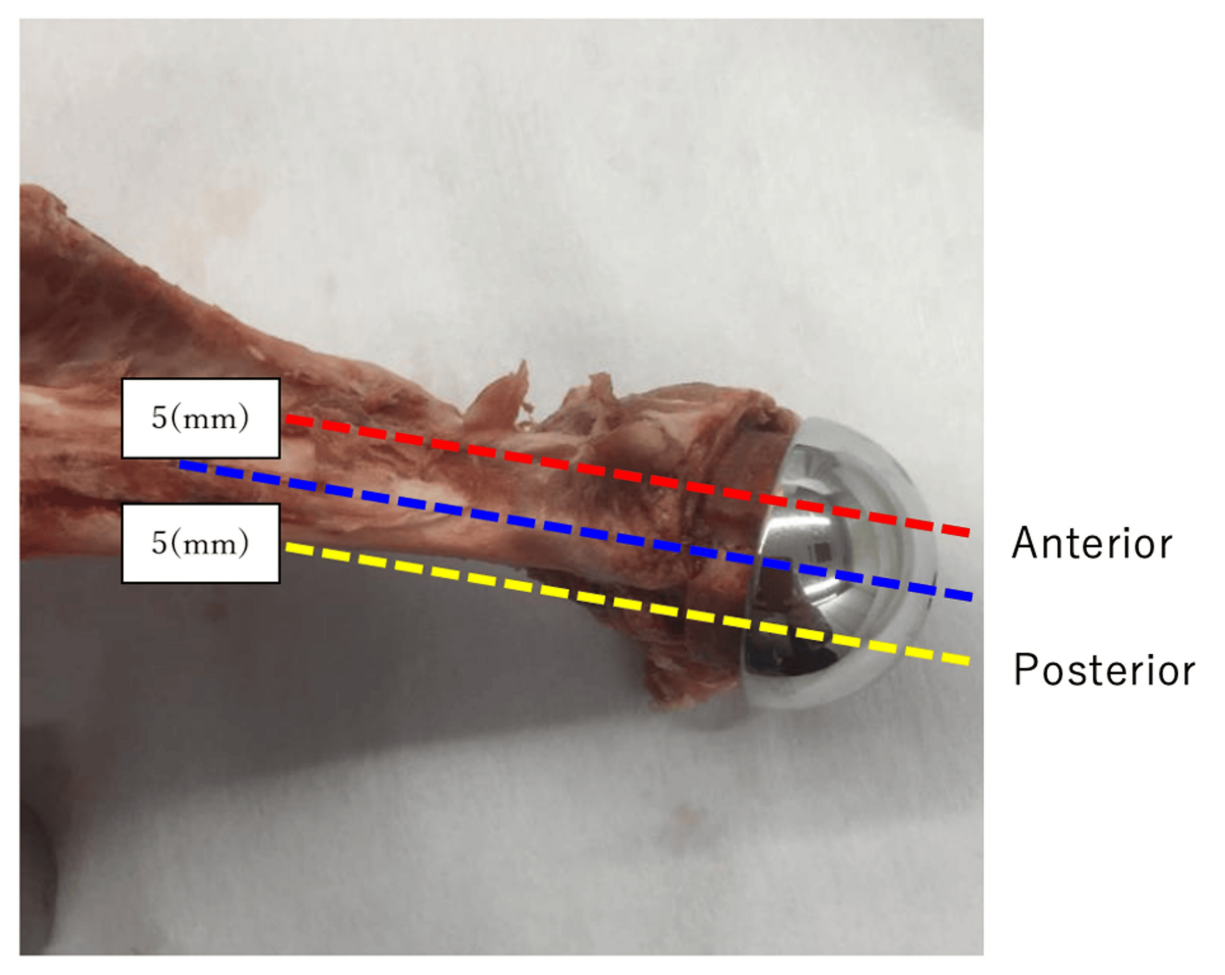
The direction and plane of arbitrary slices created with tomosynthesis of the pig scapula. The sections were taken across the anterior, posterior, and mid regions.

We conducted an ex vivo diagnostic accuracy study using a pig scapula model to compare the diagnostic performance of radiography, CT, and tomosynthesis for detecting glenoid bone defects after RSA. Four shoulder glenosphere and baseplate components (Delta XTEND®, DePuy, Warsaw, IN, USA) were implanted in the pig scapula (Figures 1, 2), leading to three different types of glenoid bone defect models. We used four slaughter pig scapula bones for our experiments.

Intact control model

For the control model, a scapula without any glenoid bone defects was selected, and the baseplate and glenosphere components were then implanted without causing damage to the bone (Figure 1a).

Anterior, inferior, and posterior glenoid bone defect models

The glenoid bone defects (mean size, 1.5 cm^3^) were created until the closest screw was completely exposed and placed on the anterior, inferior, and posterior edges of the glenoid (Figures 1b-1d). The glenoid bone defects were filled with 1% agarose to simulate granuloma tissue and reduce air artifacts around the implant that can interfere with the imaging procedures [9].

Imaging method

Three types of imaging were performed for the four pig scapulae. Radiographic evaluations (63 kV, 360 mA, 50 ms) were performed in the AP views for each scapula.

CT examinations of the scapulae were performed using a high-resolution CT scanner (Aquilion 16; Toshiba, Tokyo, Japan). An extended-scale technique was used to suppress the resulting metal artifact. Scans were taken using the following settings: 120 kV, 400 mAs, 500 ms spiral, 0.5 mm slice thickness, and a sagittal reconstruction interval of 0.5 mm. Then, the coronal and axial plane images were reconstructed, and two slices around the glenoid bone defects were acquired for each specimen.

For the novel tomographic technique (tomosynthesis), a fully digital diagnostic table with a direct-conversion-type flat panel detector was used (SONIALVISION Safire17; Shimadzu Corp., Kyoto, Japan). Seventy-four frames were acquired at a rate of 30 frames/s with a fixed X-ray condition (140 kV, 320 mA, 20 ms). These linear scans were transformed into CT images with 40° rotation using an automatic reconstruction algorithm. Projection images were separated into metal and metal-free images. The metal and metal-free reconstruction tomographic images were automatically created. Finally, these two images were combined. The anterior, medial, and posterior topographies for each specimen were acquired (Figure 2).

Sensitivity and specificity

Twelve blinded assessors, all orthopedic surgeons experienced in clinical radiographic evaluation, independently assessed anteroposterior radiographs, tomosynthesis images, and CT images. All images were printed and evaluated under identical conditions. For each image, the assessors were required to classify the findings into one of four predefined models (anterior defect, posterior defect, inferior defect, or no defect).

The presence or absence of bone defects in each model was predefined and used as the reference standard. For each imaging modality, assessments were categorized as true positive, false negative, false positive, or true negative based on agreement with the reference model. Sensitivity was defined as the proportion of correctly identified bone defect-positive models, and specificity was defined as the proportion of correctly identified bone defect-negative models. Measurements were repeated three times for each imaging modality, and sensitivity and specificity were calculated accordingly.

Statistical analysis

All analyses were performed in R (R Foundation for Statistical Computing, Vienna, Austria) [10]. Fisher’s exact test was used to compare the differences in sensitivity and specificity between each imaging method. p-values < 0.05 were considered statistically significant.

## Results

**Figure 3:**
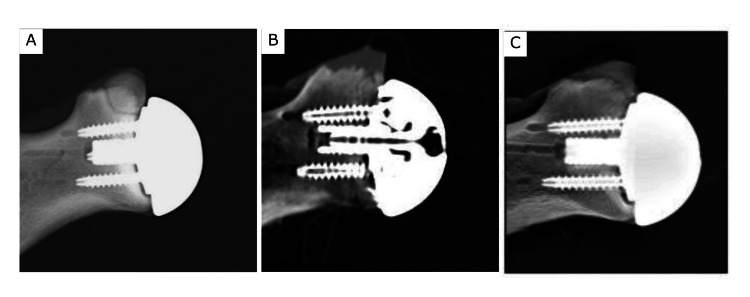
Control model a. radiographic evaluation, b. coronal CT image, and c. tomosynthesis.

For the control model, no significant differences in sensitivity and specificity were observed among radiographic evaluation, CT, and tomosynthesis (p = 0.32, p = 1) (Figure 3).

**Figure 4:**
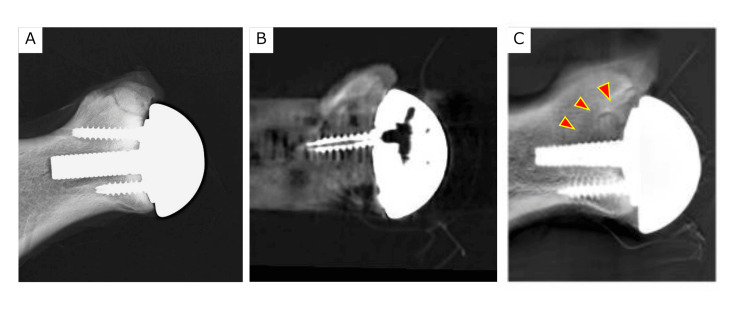
Anterior glenoid bone defect model a. radiographic evaluation, b. coronal CT image, and c. tomosynthesis. Arrowheads show areas of the anterior glenoid bone defect.

**Figure 5:**
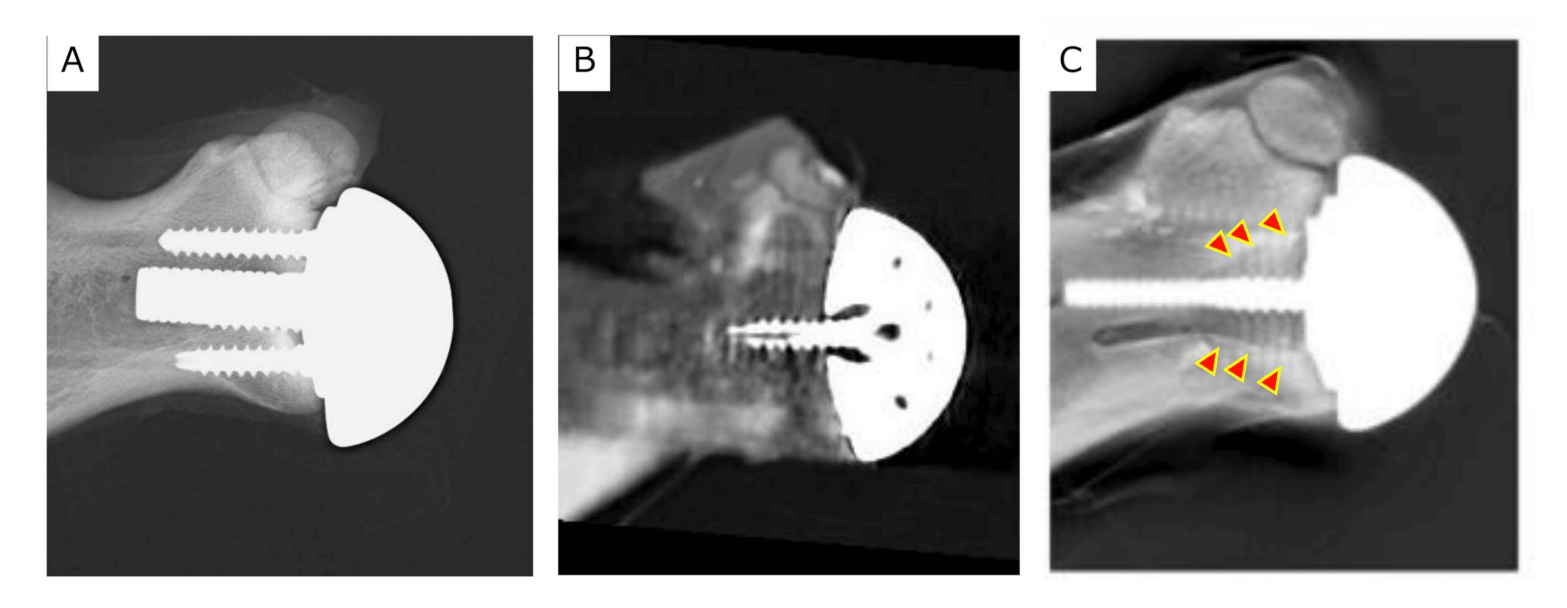
Posterior glenoid bone defect model a. radiographic evaluation, b. coronal CT image, and c. tomosynthesis. Arrowheads show areas of the posterior glenoid bone defect.

For the anterior and posterior glenoid bone defect model, the edges of the bone defects were blurred and could not be recognized clearly, despite the adoption of CT with metal reduction. However, in tomosynthesis, the superior edges of the bone defects were detectable (Figures 4, 5).

Tomosynthesis exhibited a significantly higher sensitivity than radiographic evaluation and CT (p < 0.01, p < 0.01). However, there was no significant difference in specificity among the three images (p = 0.07, p = 0.5).

**Figure 6:**
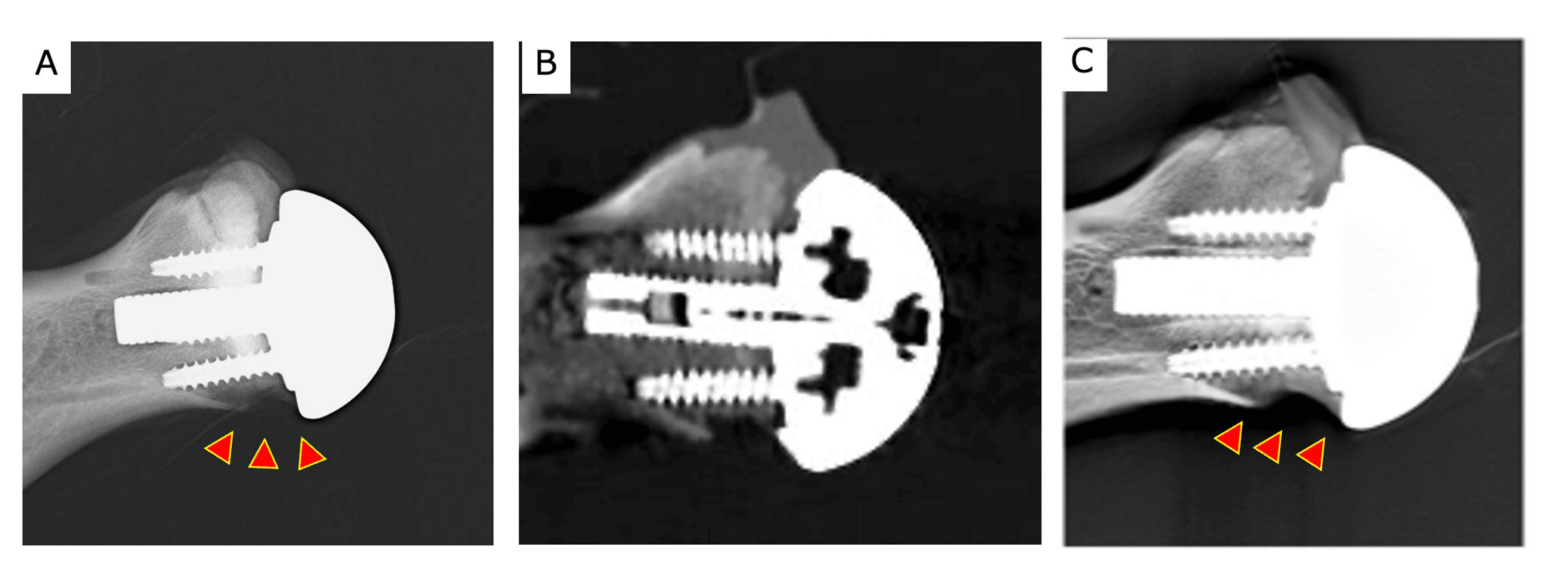
Inferior glenoid bone defect models a. radiographic evaluation, b. coronal CT image, and c. tomosynthesis. Arrowheads show areas of the inferior glenoid bone defect.

The inferior glenoid bone defect was easily observed in radiographic evaluation and tomosynthesis; however, in CT, it was difficult to detect the bone defect because of metal artifacts (Figure 6).

Consequently, both sensitivity and specificity were lower in CT than in radiographic evaluation and tomosynthesis in this study (p < 0.01, p < 0.01).

Table 1 summarizes the sensitivity and specificity data.

**Table 1:** A comparison of the sensitivity and specificity of radiographic evaluation, CT, and tomosynthesis imaging modalities. Fisher’s exact test was used to compare the differences in sensitivity and specificity. Statistical significance was defined as p < 0.05. Significant p-values are denoted by an asterisk (*).

		Sensitivity	Specificity
Control	Radiographic evaluation	41.6%	91.7%
	CT	66.7%	88.9%
	Tomosynthesis	75%	91.7%
	p-value	0.32	1
Anterior glenoid bone defect	Radiographic evaluation	25%	100%
	CT	25%	86.1%
	Tomosynthesis	83.3%	94.4%
	p-value	<0.01 *	0.07
Posterior glenoid bone defect	Radiographic evaluation	8.3%	94.4%
	CT	33.3%	86.1%
	Tomosynthesis	83.3%	94.4%
	p-value	<0.01 *	0.5
Inferior glenoid bone defect	Radiographic evaluation	100%	100%
	CT	50%	83.3%
	Tomosynthesis	100%	100%
	p-value	<0.01 *	<0.01 *

## Discussion

This study showed that anterior and posterior glenoid bone defects were not detectable by simple radiographic evaluation and CT. Tomosynthesis helped detect anterior and posterior glenoid bone defects more clearly than radiographic evaluation and CT. Impingement after RSA usually occurs in the inferior edge of the scapular neck with arm adduction, and the location of impingement is influenced by certain variables, including surgical factors such as the location and tilting angle of the glenosphere on the glenoid [11], along with implantation-related factors, such as implant size, the center of rotation offset, and the angle of the humeral neck shaft [12,13]. Generally, if the impingement continues, the glenoid bone defect might increase in size; thus, early detection is necessary. Various measures have been developed to reduce these complications. For example, in BIO-RSA [14], downward tilting of the glenosphere or achieving offset using the humerus or glenosphere are generally used in current surgeries to reduce impingement [15,16].

Additionally, a cadaveric study suggested that glenoid bone loss of the anterior side may occur due to impingement between the glenoid and humerus after RSA [17]. Unfortunately, if the defect is not detected and multiple impingements result in the growth of the glenoid bone defect, baseplate failure might occur [18]. Therefore, careful monitoring is required around the baseplate to avoid such failures. To the best of our knowledge, no previous study has focused on detecting anterior and posterior glenoid bone defects around the baseplate after RSA.

Tomosynthesis requires < 7 s and is performed in an upright position, like a radiographic evaluation, and is therefore easy to use in daily clinical practice. It also has the advantage of detecting glenoid bone defects and any changes with thin-section reconstruction. Additionally, tomosynthesis requires a relatively low radiation dose compared with CT.

Anterior and posterior glenoid bone defects could not be detected with radiographic evaluation because the bone defects and the glenoid bone overlapped. Similarly, glenoid bone defects were not clearly recognized using CT because the defects were surrounded by the baseplate and screw. Therefore, the influence of the metal artifact was greater than usual. Ferreira et al. reported that metal artifacts prevented identification of bone resorption around the baseplate of RSA, and more effective imaging techniques were required to determine if bone resorption had occurred after RSA [19]. Our study showed that tomosynthesis could create three image slices in this pig model without metal artifacts. Using these images, the glenoid bone defect and its position could be identified. Therefore, tomosynthesis provides precise information regarding the glenoid bone defect around the RSA prosthesis. Kurmis et al. and Vessely et al. reported that for detecting bone defects or bone osteolysis after total knee arthroplasty, CT and magnetic resonance imaging (MRI) should not be routinely used around the arthroplasty site, considering their cost and low sensitivity [20,21]. In contrast, tomosynthesis has an approximately 70% lower cost than MRI and CT [8]. Although the cost of tomosynthesis was slightly higher than radiographic evaluation, tomosynthesis offers much higher selectivity and sensitivity. Therefore, tomosynthesis is suitable for the detection of glenoid bone defects after RSA.

The radiation dose is also an important concern in routine checkups. The radiation dose in radiographic evaluation was small (0.9 mGy). However, this examination could not detect the anterior and posterior glenoid bone defects. The radiation dose in CT was 80 times higher than that in radiographic evaluation. The use of tomosynthesis reduced the radiation dose by 94.4% compared with CT while demonstrating higher sensitivity and specificity. Given the radiation dose, tomosynthesis may be favorable for follow-ups after RSA.

This study has some limitations. The experimental design was ex vivo in nature and relied on a pig scapula, which may not fully reproduce the postoperative anatomy, bone quality, or soft-tissue conditions of the human shoulder. Bone defects were artificially created and intentionally uniform, which simplifies the complexity and variability of glenoid bone loss encountered in clinical practice. The absence of surrounding soft tissues may have influenced image interpretation and limited direct translation to in vivo postoperative imaging. Another limitation was that the interobserver reliability was not formally assessed. However, the primary aim of this proof-of-concept study was to compare diagnostic performance across imaging modalities; as such, inter-evaluator agreement was not a primary consideration. Consequently, the small sample size and experimental nature of the study limit external validity, and the findings should be interpreted with caution. Further clinical studies with larger cohorts and in vivo conditions are warranted to validate these results.

## Conclusions

Despite its limitations and the need for further validation in clinical studies, this study highlights the potential utility of tomosynthesis imaging. Visualization of anterior and posterior glenoid bone defects was limited with radiographic evaluation and CT because of image overlap and metal artifacts. In this controlled ex vivo model, tomosynthesis enabled acquisition of arbitrary cross-sectional images with minimal metal artifacts, allowing clearer identification of glenoid bone defects. Although these findings are limited to an experimental setting, tomosynthesis may aid in characterizing the presence and distribution of anterior and posterior glenoid bone defects that could be underestimated by conventional imaging.
